# Eye movements and user emotional experience: a study in interface design

**DOI:** 10.3389/fpsyg.2025.1455177

**Published:** 2025-03-19

**Authors:** Ningna Sun, Yufei Jiang

**Affiliations:** Department of Industrial Design, School of Art, Jiangsu University, Zhenjiang, Jiangsu, China

**Keywords:** eye-tracking, user emotional experience, pleasure-arousal-dominance model, interface design, cycling app

## Abstract

The purpose of this study is to explore the correlation between eye movement metrics and user emotional experience metrics during the user’s process of using the interface in a task-oriented manner through an eye-tracking study. Fifty-four participants were recruited, who were divided into two groups and asked to complete the same task using two different sets of interfaces. The two sets of interfaces were proved to have differences in the emotional experience of users before the experiment. The participants’ eye movement data were recorded as they operated, and correlation analyzes were performed using biserial correlation tests. The results show that different interface designs affect the three dimensions of user emotional experience (PAD) and also lead to changes in eye movement patterns as the users complete tasks. Interface designs that elicit higher pleasure will lead to longer duration of fixations. Interface designs that elicit higher arousal will lead to more fixations and higher peak velocity of saccades. Interface designs that elicit higher dominance will lead to longer duration of fixations, fewer fixations and fewer saccades. This study identifies eye movement metrics related to the user emotional experience in interface design that are different from those in other fields, providing a new perspective for the scientific validation of the emotional experience in interface design. The designers can apply the findings to optimize the specific interface design to improve the user’s emotional experience.

## 1 Introduction

Nowadays, the experience economy has gradually become the mainstream of our time (Pine and Gilmore, 1999). User experience has become a popular topic in interface design in recent years. User experience focuses not only on the functionality provided by the interface itself, but also on the emotional experience of users. User emotional experience is a crucial part of the user experience. According to Schmitt (1999), user experience can be divided into five modules: sense, feel, think, act, relate, with the feel module representing the emotions and feelings that are generated by exposure to things. Daniel (2000) defined user experience as what a person did, thought, and felt when operating or working with a product or service, which also includes the user emotional experience. Hassenzahl and Tractinsky (2006) summarized user experience as a consequence of a user’s internal state (predispositions, expectations, needs, motivation, mood, etc.), the characteristics of the designed system (e.g., complexity, purpose, usability, functionality, etc.) and the context (or the environment) within which the interaction occurs (e.g., organizational/social setting, meaningfulness of the activity, voluntariness of use, etc.). The user emotional experience is a type of internal state. Law and Van Schaik (2010) emphasized that user experience does not only include usability, but also other cognitive, socio-cognitive and affective aspects of users’ experience in their interaction with artifacts, placing a greater importance on user emotional experience. ISO 9241-210 officially defined that user experience includes all the users’ emotions, beliefs, perceptions, physical and psychological responses, behaviors and accomplishments that occur before, during and after use (ISO, 2010). This definition still emphasizes the importance of the user emotional experience within it.

The ambiguous and elusiveness of the user emotional experience presents a significant challenge in conducting validation studies on it (Law et al., 2014). Traditional research on user emotional experience uses subjective scales based on self-report [e.g., PAD emotional scale (Mehrabian and Russell, 1974), SAM (Bradley and Lang, 1994), PANAS (Cotigă, 2012), Emocards (Desmet et al., 2001), etc.], which enable rapid access to reliable emotional information at a low cost. Objective measurements based on physiological signals, such as visual signals, have emerged in recent years (Ahmad and Khan, 2022; Zhang et al., 2023). However, they are still immature due to the high experimental costs, high barriers to using the equipment, and the possible impact on the user emotional experience from wearing the equipment. Nevertheless, changes in physiological signals are objectively measurable and offer irreplaceable advantages in studying user emotional experience.

The directness, real-time, non-invasiveness and wide application of eye-tracking technology makes it a very popular method of objective study of user emotional experience now (Klaib et al., 2021; García and Cano, 2022). At the same time, the eye-tracking experiment is also an important experimental method in interface design research, which can reflect specific problems in interface design and provide valuable data and insights, thus helping designers to optimize the interface design and provide a better user emotional experience (Ellis et al., 1998). Therefore, the use of eye-tracking experiments in interface design to study user emotional experience has great potential.

Scholars have drawn conclusions from existing eye-tracking studies on user emotional experience (Mele and Federici, 2012; Skaramagkas et al., 2021). These studies have explored the possible correlation between eye movement metrics and specific metrics of user emotional experience, but have not yet established a comprehensive system for evaluating the user emotional experience. Moreover, most experimental stimulus in such studies are pictures or videos, with very few utilizing the interaction process as a experimental stimuli (Lim et al., 2020; Skaramagkas et al., 2023). No specific conclusions have been drawn in the field of interface design. Therefore, the focus of this study is to explore the correlation between eye movement metrics and user emotional experience metrics during task-oriented use, with the aim of offering new insights for evaluating and enhancing user emotional experiences in interface design.

The idea of this study involves: studying related literature to provide theoretical references for metric selection and to formulate hypotheses; conducting an eye-tracking experiment to obtain quantitative data on user emotional experience and eye movement during the participants’ interface use; carrying out data processing and analysis to explore the specific correlation between eye movement metrics and the user emotional experience metrics during interface use; and discussing the significance and limitations of this study, as well as proposing further research directions.

## 2 Hypotheses

### 2.1 Basis for hypotheses

Existing studies on user emotional experience typically identify the research metrics based on the theoretical foundations of emotion in psychology. Watson et al. (1988) categorized user emotional experience into two mutually independent metrics: Positive Affect and Negative Affect, which are similar to the Positive and Negative metrics proposed by Desmet (2018). Russell (1980), on the other hand, classified the user emotional experience into two dimensions, Valence and Arousal. Desmet et al. (2001) proposed similar metrics of pleasantness and arousal. The theories of Bradley and Lang (1994) and Mehrabian (1995) proposed three metrics: Pleasure, Arousal, and Dominance, offering a more refined way of categorizing the metrics of user emotional experience.

The three metrics of user emotional experience, pleasure, arousal and dominance (abbreviated as PAD), are based on environmental psychology. These metrics focus on the individual’s emotional response to a variety of stimuli in specific environments, which can fully capture the user’s emotional changes (Mehrabian and Russell, 1974). Therefore, the three PAD metrics are commonly used in applied research to evaluate user emotional experience (Pine and Gilmore, 1999). Using these three metrics, Petermans et al. (2009) studied user emotional experience in retail shop environments; Tantanatewin and Inkarojrit (2018) analyzed the effect of colors on user emotional experience in a restaurant atmosphere. Yang et al. (2022) integrated the Pleasure-Arousal-Dominance (PAD) Emotion Model and the Five Factor Model (FFM) to examine the influence of three key factors on user emotional experience in mobile libraries: specifically, user interface, interaction quality, and service environment. In these user emotional experience studies based on specific situations, the three PAD metrics explain the differences in user emotional experience in more detail. Consequently, scholars have begun to use these PAD metrics to explore user emotional experience in interface design. A smooth interface operation process, without additional frustrations and challenges, is expected to enhance the user emotional experience. In such cases, the user emotional experience will be reflected as higher pleasure, lower positive arousal, and higher dominance across the three PAD metrics (Tian et al., 2008).

### 2.2 Contents for hypotheses

The eye-tracking experiment is now recognized by academics as a method for evaluating emotional experiences. Some scholars have conducted studies using stimulus such as pictures and videos and have drawn conclusions regarding the correlation between eye movement metrics and user emotional experience on the three PAD metrics. Therefore, in the field of interface design, there may also be a correlation between eye movement metrics and user emotional experience metrics (PAD) when the interaction process serves as the stimuli material during user interaction with the interface. Following the preceding discussion, the subsequent research hypothesis has been delineated:

*H0*: Different interface designs affect the three dimensions of user emotional experience (PAD), which in turn affect eye movement patterns as users complete tasks.

#### 2.2.1 Eye movement metrics and pleasure

Pleasure indicates the positive or negative characteristics of an individual’s emotional state, signifying the degree to which an individual feels pleasure or displeasure (Mehrabian and Russell, 1974). Just and Carpenter (1976) found that prolonged gaze may indicate that the target stimuli is highly attractive and of great interest to the user, leading to further increases in user pleasure. This notion has also confirmed in recent studies by Zhang et al. (2024) and Vehlen et al. (2024) in interaction scenarios involving diverse modalities. Pupil size is regulated by the autonomic nervous system (Andreassi, 2010), and there is a strong link between the generation of emotion and the autonomic nervous system (Ekman et al., 1983). People are more sensitive to negative emotional experiences and are more likely to undergo pupil dilation than positive emotional experiences (Babiker et al., 2013; Derksen et al., 2018; Oliva and Anikin, 2018). Following the preceding analysis, the subsequent research hypotheses have been delineated:

*H1a*: Interface designs that elicit higher pleasure will lead to longer duration of fixations when users complete tasks.

*H1b*: Interface designs that elicit higher pleasure will lead to smaller pupil diameters when users complete tasks.

#### 2.2.2 Eye movement metrics and arousal

Arousal indicates an individual’s level of neurophysiological activation, signifying an individual’s level of emotional arousal (Mehrabian and Russell, 1974). Henderson and Ferreira (1990) found that a rise in the difficulty of reading material led to an increase in the number of fixations, suggesting that high workloads lead to a higher number of fixations. Ward and Marsden (2003) observed that high workloads can also result in an increase in arousal. Thus, there may also be a correlation between the number of fixations and arousal. Zsidó (2024) has also found that stimuli that produce emotional arousal attract and hold attention, which is often manifested as an increase in the number of fixations. Pupil diameter and emotional arousal are suggested to be correlated, as they are both associated with the activation of the autonomic nervous system (Mathôt, 2018). It is widely acknowledged that pupil size typically corresponds with increasing levels of arousal (Beatty and Lucero-Wagoner, 2000), a relationship that has been consistently corroborated in subsequent empirical investigations (Nassar et al., 2013; Mcgarrigle et al., 2017). Galley (1993, 1998) found a correlation between saccadic velocity and arousal. He concluded from a series of studies on driving that lower arousal corresponded to lower saccadic velocity, whereas increased arousal resulted in faster saccadic velocity. The average peak velocity of saccades is an important metric for evaluating emotions within saccadic velocity metrics, with significant potential applications in ergonomics and other related fields (Di Stasi et al., 2013). Following the preceding analysis, the subsequent research hypotheses have been delineated:

*H2a*: Interface designs that elicit higher arousal will lead to more fixations when users complete tasks.

*H2b*: Interface designs that elicit higher arousal will lead to larger pupil diameters when users complete tasks.

*H2c*: Interface designs that elicit higher arousal will lead to higher peak velocity of saccades when users complete tasks.

#### 2.2.3 Eye movement metrics and dominance

Dominance indicates the strength of an individual’s sense of control over external situations or others, focusing on an individual’s emotional responses to various stimuli within a specific context (Mehrabian and Russell, 1974). Ehmke and Wilson (2007) noted that users would feel disoriented if the interface lacks expected information or contains misleading elements with unclear meanings. Consequently, this reduction in dominance leads to an increase in the number of short gazes and a decrease in the average duration of fixations. Interfaces that are inefficient in search usually provide a poor user emotional experience, which makes it difficult for the user to feel in control of the interface. This difficulty is reflected in eye movement behaviors, characterized by the higher number of fixations and saccades (Goldberg and Kotval, 1999; Scott and Hand, 2016; Tzafilkou and Protogeros, 2017). And when the interface contains more meaningful visual cues, search efficiency improves, which reflected in eye movement behaviors as an increase in the amplitude of saccades (Goldberg et al., 2002). Following the preceding analysis, the subsequent research hypotheses have been delineated:

*H3a*: Interface designs that elicit higher dominance will lead to longer duration of fixations when users complete tasks.

*H3b*: Interface designs that elicit higher dominance will lead to fewer fixations when users complete tasks.

*H3c*: Interface designs that elicit higher dominance will lead to fewer saccades when users complete tasks.

*H3d*: Interface designs that elicit higher dominance will lead to larger amplitude of saccades when users complete tasks.

## 3 Methods

### 3.1 Experimental design

The purpose of this experiment is to explore the correlation between eye movement metrics and user emotional experience metrics during task-oriented use. Since binary classification of emotional experience has been shown to be more effective and accurate than multi-class classification in emotional experience research using eye-tracking experiments (Alghowinem et al., 2013; Al-gawwam and Benaissa, 2018). Therefore, in this study, the user emotional experience was transformed into a binary variable that was categorized into high and low levels. The participants were divided into two groups and interacted with two different sets of interfaces to ensure that the user emotional experience of the two groups were at different levels on the three PAD metrics. Before the formal experiment, the self-report method was used to verify differences between the two sets of interfaces in the three metrics of PAD for user emotional experience. Then an eye-tracking experiment was used to explore the relationship between eye movement metrics and user emotional experience metrics.

### 3.2 Participants

Fifty-four participants were recruited for this experiment, 34 males and 20 females (Mean age = 22.556 years, SD = 2.530, range = 18–29). All participants possessed either normal or corrected-to-normal vision. They were randomly allocated into two equal groups, designated Group A and Group B, consisting of 27 individuals per group.

### 3.3 Equipment

**Figure 1:**
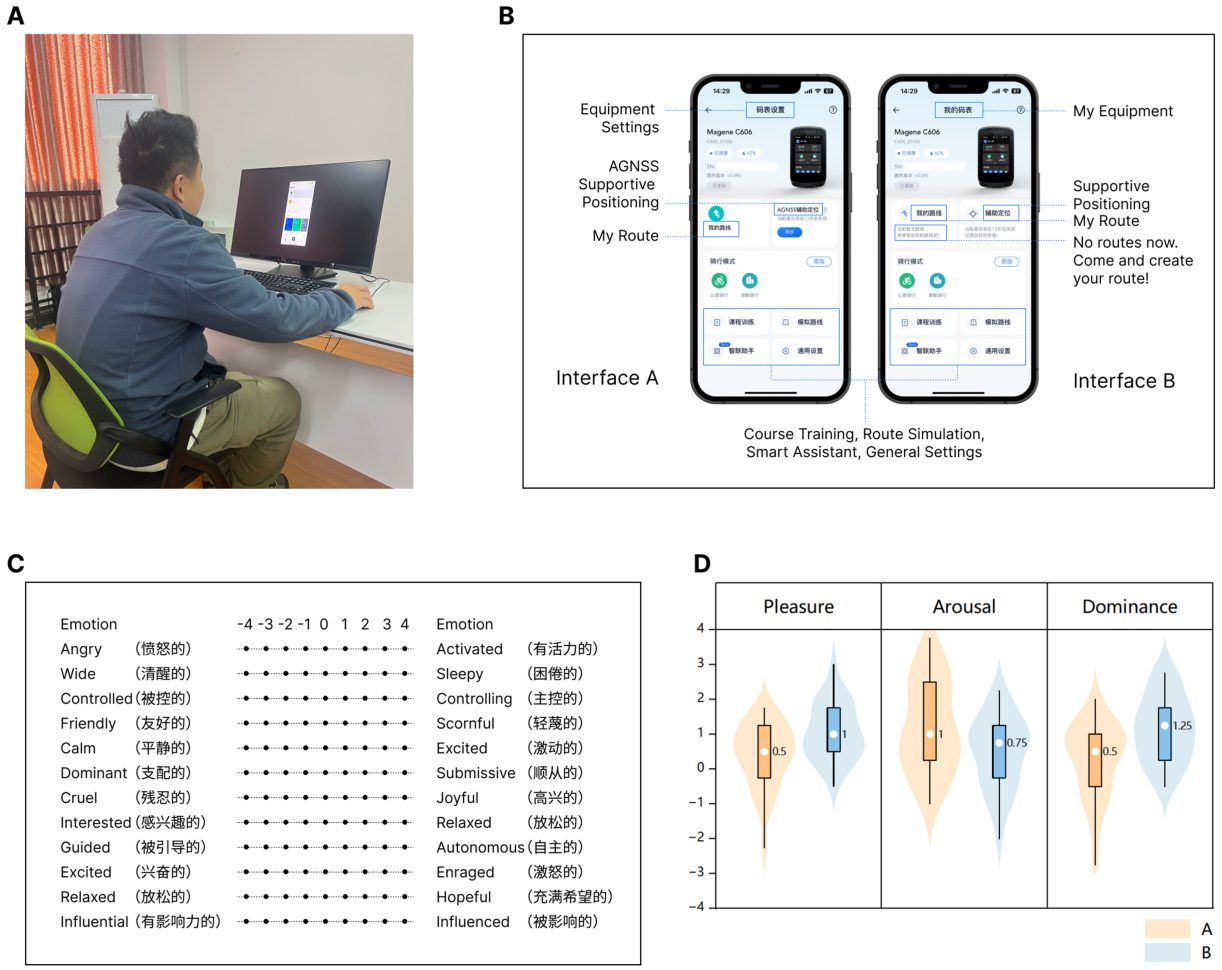
**(A)** Example of experimental setup. Participants were seated in front of a monitor to operate the interface, and the eye-tracker was mounted on the lower edge of the monitor. The positions of the tables and chairs used for the experiment were fixed to ensure that the distance between each subject and the screen was the same. There was no direct sunlight in the laboratory and the lighting environment was kept consistent. **(B)** Examples of Interface A and Interface B. Examples of Interface A and Interface B. In this step, the participants needs to click “My Route” in the interface to complete the next step of setting up the route. There are three differences in this example. Firstly, the heading “My Devices” in Interface B is more friendly and accessible than the heading “Device Settings” in Interface A. Secondly, Interface B provides the task-related friendly reminder element “No route now. Come and create your route!,” which is missing in Interface A. Thirdly, the icon and card style of “My Routes” in Interface B is consistent with the style on the right (AGNSS Supportive Positioning) and at the bottom (Course Training, Route Simulation, Smart Assistant, and General Settings), while the icon and card styles in Interface A are more confusing. **(C)** 12-item PAD emotional scale. **(D)** User emotional experience values for Interface A and Interface B obtained using the self-report method.

Integrated within a 27-inch monitor, a Tobii Pro Fusion eye-tracker was deployed for eye movement capture. Operating at a 60 Hz sampling rate with a spatial resolution below 0.5°, the eye-tracker ensured accurate tracking. The Tobii Pro Lab software facilitated the recording, analysis of the eye-tracking data, and the calibration process. Participants were approximately 65 cm away from the screen. The laboratory was shielded from direct sunlight and illuminated with fluorescent lamps to ensure consistent lighting conditions within the experimental environment. The experimental setup is illustrated in Figure 1A.

### 3.4 Materials

The widely used cycling app in China, Onelap, served as the foundation for creating experimental materials. In accordance with this study, two sets of high-fidelity interface prototypes, labeled A and B, were created, specifically designed for the route setting function. The text of interface was in Chinese, as it is the native language of the participants. Interface A, operated by Group A, which lacked helpful visual elements and logical visual style, was designated as the low user emotional experience group, while Interface B, operated by Group B, which had better design of visual elements and visual style, was designated as the high user emotional experience group. Throughout the experiment, participants were required to use the interface to complete a route-setting task, which helps to investigate the correlation between eye movement metrics and user emotional experience metrics as users engage in specific tasks with the interface. The interface prototypes were created in Figma and then imported as stimulus materials into the Tobii Pro Lab software, enabling users to operate them on a computer. Sample visual displays are presented in Figure 1B.

To verify the differences in user emotional experience between interfaces A and B, we first employed the traditional self-report method to subjectively measure the user emotional experience for both sets of interfaces. We recruited 50 informal experimental participants and randomly distributed them into two groups of 25 each. They were asked to operate Interface A or B and to rate the emotional experience of the interfaces using the PAD emotional scale, which is commonly used in interface design (Mehrabian, 1995; Xiaoming et al., 2008). This scale is constituted by a 9-point semantic differential Likert scale comprising 12 pairs of adjectives denoting disparate emotional states, with 4 pairs of adjectives in each of the three dimensions of the PAD. The mean value of all items under each of the three PAD dimensions is the final score value for that dimension (see Figure 1C).

Shapiro–Wilk (S–W) tests were used to conduct a normality test on the user emotional experience data. The *p*-values for pleasure (*p* = 0.382), arousal (*p* = 0.690), and dominance (*p* = 0.177) all exceeded the threshold of 0.05, indicating that they all satisfied the normal distribution. Next, tests for homogeneity of variances were conducted on the user emotional experience data. The p-values for pleasure (*F* = 0.107, *p* = 0.745), arousal (*F* = 1.915, *p* = 0.173), and dominance (*F* = 0.556, *p* = 0.177) all exceeded the threshold of 0.05, indicating that they all satisfied the homogeneity of variances.

Independent samples *t*-tests were implemented to analyze the user emotional experience data (see Figure 1D). The analysis results are presented in Table 1. Participants using Interface A (*M* = 0.38, SD = 0.955) had significantly less pleasant than those using Interface B (*M* = 1.07, SD = 0.843) (*t* = −2.708, *p* = 0.009 < 0.05). Participants using Interface A (*M* = 1.2, SD = 1.337) had significantly more arousal than those using Interface B (*M* = 0.47, SD = 1.064) (*t* = 2.137, *p* = 0.038 < 0.05). Participants using Interface A (*M* = 0.22, SD = 1.14) had significantly less dominance than those using Interface B (*M* = 1.16, SD = 0.968) (*t* = −3.144, *p* = 0.003 < 0.05). This proved a significant difference in user emotional experience between the two groups, with the user emotional experience of Interface B being superior to that of Interface A.

**Table 1:** Data from the results of independent samples *t*-tests of the user emotional experience.

		*M*	SD	*t*	*p*
Pleasure	A	0.38	0.955	−2.708	0.009**
	B	1.07	0.843		
Arousal	A	1.2	1.337	2.137	0.038*
	B	0.47	1.064		
Dominance	A	0.22	1.14	−3.144	0.003**
	B	1.16	0.968		

M, mean; SD, standard deviation.

*T-value, P-value from independent samples t-tests.*

*p < 0.05, **p < 0.01.

### 3.5 Procedures

In the preliminary phase of the experiment, researchers meticulously inspected all materials and equipment to confirm their operational readiness, while also verifying the completeness of the experimental guidelines and participant questionnaires. With all preparations in place, researchers then provided a comprehensive briefing to the participants, detailing the experiment’s goals, procedures and important considerations, and responded to any questions participants posed. After participants fully understood and agreed to the experimental protocol, they were asked to sign an informed consent form. Subsequently, participants completed a pre-experiment questionnaire to provide basic demographic information. After completing the questionnaire, they carefully read the instructions for the experimental task, which required them to complete the following tasks: create a new cycling route in the APP with the starting and ending points both set to Baima Park. And add two waypoints to the route, the first waypoint is Wangjiawan and the second waypoint is Zijinshan Road. Save the route to the app after previewing and confirming its accuracy. The researcher informed the subjects that there was no need to memorize the tasks as they would be prompted again before starting the task.

Before the formal experiment, participants were asked to sit in front of the eye-tracker and adjust to a comfortable posture, ensuring that the distance between all participants and the monitor was consistent. After adjusting their posture, participants underwent a 9-point calibration in front of the eye-tracker.

The formal experiment commenced following the completion of calibration. The researcher reiterated the experimental tasks to the participants, ensuring that they had a thorough understanding of the task requirements before declaring the official start, at which point the participant could initiate their operations. Throughout the participant’s engagement with the task, the researcher maintained an observation of the participant’s eye movements to guarantee that their eyes remained within the operational range of the eye-tracker. The researcher refrained from responding to any inquiries from the participants during the task execution and abstained from influencing the participant’s approach to the process. Upon the participant’s belief that the task had been completed, they would seek confirmation from the researcher, who would then verify and officially announce the task’s completion.

## 4 Results

After excluding samples with insufficient collection rates for meaningful data analysis, 50 valid samples were eventually obtained. The relevant eye-tracking data were exported through the eye-tracker. The level of pleasure, arousal and dominance of user emotional experience were transformed into binary variables (1 means high level, 0 means low level). Biserial correlation tests were used to analyze the correlation between eye movement metrics and user emotional experience metrics. After obtaining the biserial correlation coefficients, the significance of the coefficients was tested.

### 4.1 Normality test

Shapiro–Wilk (S–W) tests were used to conduct a normality test on the eye-tracking data. The *p*-values for the average duration of fixations (*p* = 0.364), the average fixation pupil diameter (*p* = 0.757), the average peak velocity of saccades (*p* = 0.177) and the average amplitude of saccades all exceeded the threshold of 0.05, indicating that they all satisfied the normal distribution. The *p*-value for the number of fixations was 0.026, and when considering the kurtosis (1.241) and skewness (0.934), these values suggest that the data approximate a normal distribution. The *p*-value for the number of saccades was 0.003, and when considering the kurtosis (3.092) and skewness (1.247), these values suggest that the data approximate a normal distribution. Therefore all data can be analyzed by biserial correlation test.

### 4.2 Correlation analysis between eye movement metrics and pleasure

At different levels of pleasure, the average duration of fixations is moderately positively correlated with pleasure (*r* = 0.524), providing support for hypothesis H1a. The correlated between the average fixation pupil diameter and pleasure is very weak (*r* = 0.030) and the *p*-values is greater than 0.05 (*p* = 0.836). Therefore, there is no correlation between the average fixation pupil diameter and pleasure, hypothesis H1b is not supported.

### 4.3 Correlation analysis between eye movement metrics and arousal

At different levels of arousal, the number of fixations is moderately positively correlated with arousal (*r* = 0.503), the average peak velocity of saccades is weakly positively correlated with arousal (*r* = 0.415), providing support for hypotheses H2a and H2c. The correlated between the average fixation pupil diameter and arousal is very weak (*r* = −0.030) and the *p*-values is greater than 0.05 (*p* = 0.836). Therefore, there is no correlation between the average fixation pupil diameter and arousal, hypothesis H2b is not supported.

### 4.4 Correlation analysis between eye movement metrics and dominance

At different levels of dominance, the average duration of fixations is moderately positively correlated with dominance (*r* = 0.524), the number of fixations is moderately negatively correlated with dominance (*r* = −0.503), the number of saccades is weakly negatively correlated with dominance (*r* = −0.465), providing support for hypotheses H3a, H3b and H3c. The correlated between the average amplitude of saccades and dominance is very weak (*r* = −0.119) and the *p*-values is greater than 0.05 (*p* = 0.409). Therefore, there is no correlation between the average amplitude of saccades and dominance, hypothesis H3d is not supported.

**Figure 2:**
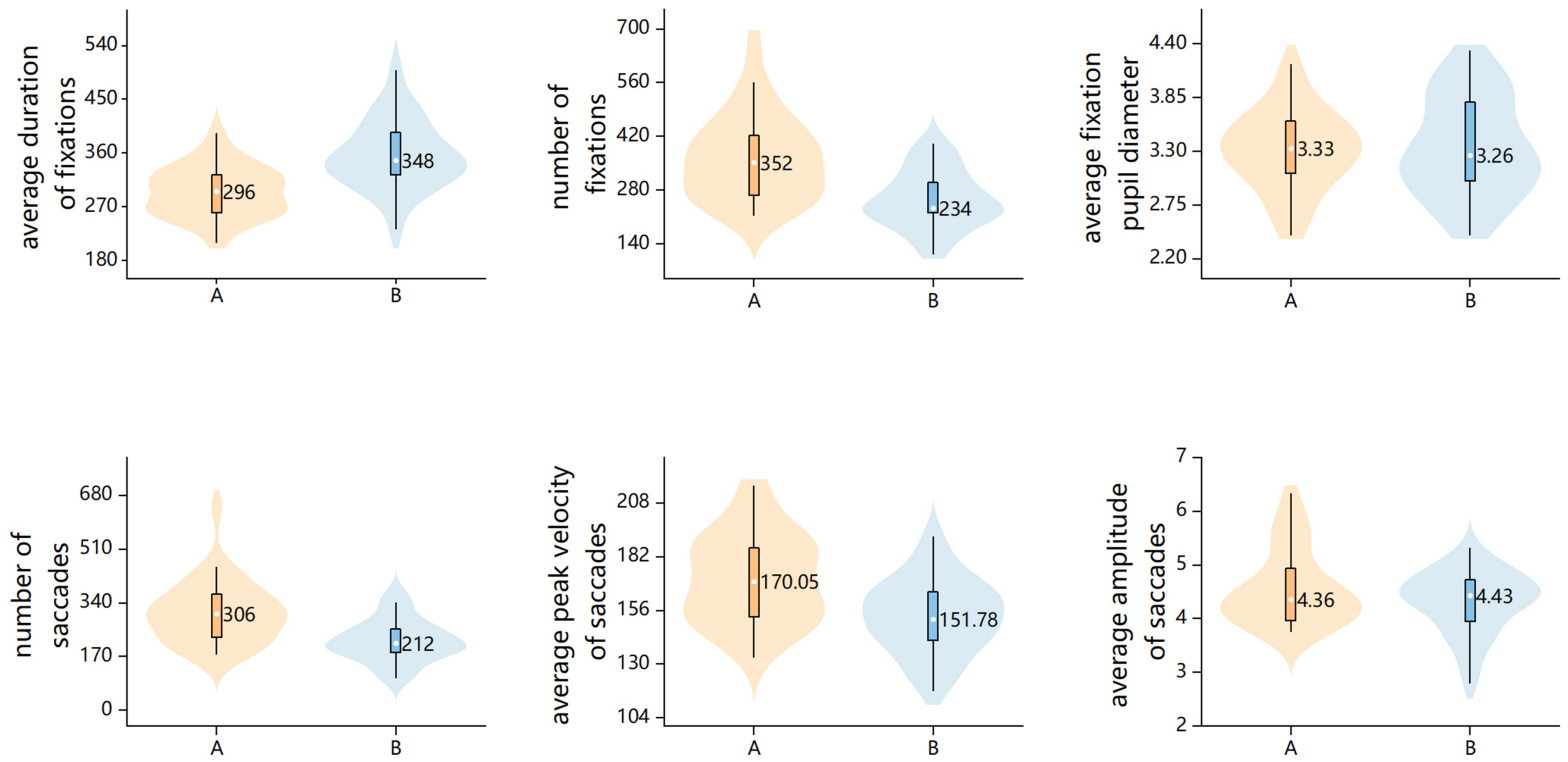
Analysis of eye movement data from Interface A and Interface B.

Based on the aforementioned analysis (see Table 2 and Figure 2), it can be observed that during the process of operating the interface to complete a task, some eye movement metrics have a certain correlation with user emotional experience metrics (PAD), thus the hypothesis H0 is supported.

**Table 2:** Data on the correlation between eye movement metrics and user emotional experience metrics (PAD).

		*r*	*p*
Pleasure	Average duration of fixations	0.524	<0.001***
	Average fixation pupil diameter	0.030	0.836
Arousal	Number of fixations	0.503	<0.001***
	Average fixation pupil diameter	−0.030	0.836
	Average peak velocity of saccades	0.415	0.003**
Dominance	Average duration of fixations	0.524	<0.001***
	Number of fixations	−0.503	<0.001***
	Number of saccades	−0.465	<0.001***
	Average amplitude of saccades	−0.119	0.409

r, biserial correlation coefficient.

*P-value from correlation coefficient tests.*

*p < 0.05, **p < 0.01, and ***p < 0.001.

## 5 Discussion

This study investigated the correlation between eye movement metrics and user emotional experience metrics during task-oriented interactions with interfaces. The findings indicate that interface designs eliciting varying levels of pleasure, arousal, and dominance indeed produce distinct patterns of eye movement. The specific findings of the study are delineated as follows:

Regarding the correlation between eye movement metrics and pleasure, this study discovered that interface designs that elicit higher pleasure will lead to longer duration of fixations. The experimental results revealed a moderate positive correlation between the average duration of fixations and pleasure, aligning with the findings of Just and Carpenter (1976), Zhang et al. (2024) and Vehlen et al. (2024). This supports the notion in interface design that sustained attention to interface elements may indicate higher interest and pleasure in the stimuli. However, this study did not find a correlation between pupil size and pleasure, which is inconsistent with previous findings (Babiker et al., 2013; Derksen et al., 2018; Oliva and Anikin, 2018). The inconsistency potentially stems from the fact that positive emotional experiences may also induce a certain degree of pupil dilation (Partala and Surakka, 2003).

Regarding the correlation between eye movement metrics and arousal, this study discovered that interface designs that elicit higher arousal will lead to an increased number of fixations and higher peak velocity of saccades. The experimental results indicate a moderate positive correlation between the number of fixations and arousal. This finding corroborates the established findings of Henderson and Ferreira (1990), Ward and Marsden (2003), and Zsidó (2024), further elucidating the robust association between heightened arousal, increased workload, and a greater number of fixations during interface interaction. Additionally, the observed weak positive correlation between the average peak velocity of saccades and arousal aligns with the findings of Galley (1993, 1998). This extends Galley’s previous conclusions, which were initially drawn in the context of driving, to the realm of interface design. However, the study did not detect a correlation between pupil size and arousal, which is inconsistent with the findings of Nassar et al. (2013) and Mcgarrigle et al. (2017). The inconsistency potentially stems from the fact that variance in user arousal during interface operation is insufficient to elicit a measurable change in pupil size. Furthermore, the absence of significant changes in pupil size can be attributed to the consistent exposure of the user’s eyes to high luminance during interface use, as the results of Pan et al. (2022, 2024) showed that the effect of arousal on pupil size was not as pronounced at high luminance as it was at low-to-mid luminances.

Regarding the correlation between eye movement metrics and dominance, this study discovered that interface designs that elicit higher dominance will lead to longer duration of fixations, fewer fixations and fewer saccades. The experimental results revealed a moderate positive correlation between the average duration of fixations and dominance. This corroborates the findings of a previous study by Ehmke and Wilson (2007), which confirmed that interfaces that bring about lower dominance elicit more short gazes. Additionally, this study found a moderate positive correlation between the number of fixations and dominance, as well as a weak negative correlation between the number of saccades and dominance. These findings are consistent with the conclusions drawn by Goldberg and Kotval (1999), Scott and Hand (2016), Tzafilkou and Protogeros (2017), which demonstrated that interface designs with ineffective search result in decreased dominance, thereby impacting the number of fixations and saccades. However, the average amplitude of saccades and dominance did not find a correlation in this study, which is inconsistent with previous findings of Goldberg et al. (2002). The inconsistency could potentially be attributed to the limited size of the experimental sample, coupled with significant inter-individual variability in the amplitude of saccadic eye movements (Abdi Sargezeh et al., 2019; Luke et al., 2018).

### 5.1 Implications

The findings of this study reveal a significant correlation between eye movement metrics and user emotional experience metrics during task-oriented interface interactions. These findings offer novel insights into the evaluative of user emotional experience within the domain of interface design. Specifically, traditional eye-movement metrics in the field of interface design, including duration of fixations, number of fixations, number of saccades, velocity of saccades, have been endowed with new meanings. These metrics serve not only as metrics of cognitive processing (Rayner, 1998), but also play crucial roles in assessing the user emotional experience.

In contrast to findings in other research areas, metrics such as pupil size, which are widely recognized as strongly correlated with emotion (Partala and Surakka, 2003), have not shown the significant correlation with the user emotional experience in interface design studies due to the variations in the experimental materials and environments utilized across these studies. Such findings imply that the correlation between eye movement metrics and user emotional experience metrics may differ across various application domains. Consequently, there is a need for more focused research to elucidate the specific mechanisms underlying the relationship between eye movement metrics and user emotional experience metrics within the realm of interface design.

The findings of this study offer innovative insights into the scientific validation of the user emotional experience in interface design. They indicates that objective data from physiological signals, such as eye movement data, can be used to assess and validate user emotional experience more accurately by researchers. The implementation of this methodology enhances the precision with which user emotional experience can be evaluated, thereby providing a scientific foundation for the enhancement of interface design.

The eye-tracking experiment is frequently employed as a valuable tool in the field of interface design to identify and solve specific design issues (Ehmke and Wilson, 2007; Joachims, 2002). The correlation between eye movement metrics and user emotional experience metrics, as demonstrated in this study, offers a valuable reference for designers. Designers may utilize these findings to inform the optimization of interface design during the actual design process, thereby enhancing the emotional experience of users.

Moreover, the findings of this study are not limited to the studied user interfaces, but can be applied to other types of functional user interfaces, providing an effective tool to validate user emotional experience in these interfaces. This finding indicates that the results of the present study possess broad applicability and can offer valuable insights for the development of functional interfaces across various domains.

### 5.2 Limitations and further suggestions

Although the study has made contributions, it also presents specific limitations necessitating additional contemplation for subsequent studies. Firstly, the recruitment of participants was not sufficiently broad to account for the differences observed between age groups. Therefore, future research could concentrate on differences in the performance between age groups during interactions. Secondly, this study currently explores the correlation between eye movement metrics and user emotional experience metrics. Further research could investigate the establishment of emotion models, enhance the evaluation system for user emotional experience, and clarify the potential for influencing user emotional experience through specific adjustments in interface design. In addition, future research could investigate the correlation between other physiological metrics, such as heart rate and electrical skin signals, and the user’s emotional experience in the context of interface design.

## Data Availability

The original contributions presented in the study are included in the article/Supplementary material, further inquiries can be directed to the corresponding author.
